# Influence of cryopreservation on drug responses and gene expression of AML cells: Implications for the use of biobanked tissues

**DOI:** 10.1111/bjh.18557

**Published:** 2022-11-10

**Authors:** Noemi Meszaros, Karin Lind, Robert Sehlke, Bojan Vilagos, Nikolaus Krall, Gregory I. Vladimer, Heinz Sill

**Affiliations:** ^1^ Exscientia Vienna Austria; ^2^ Division of Haematology Medical University of Graz Austria

The use of primary material from patients with haematological malignancies as an ex vivo model system for basic and translational research is becoming increasingly popular as researchers continue to understand translational limitations associated with tools like cell lines or organoids.^1^, ^2^, ^3^, ^4^, ^5^, ^6^, ^7^, ^8^ Importantly, primary samples contain cancer as well as stroma and immune cells allowing for the interrogation of multi‐cellular processes and interactions.^9^

Previously, our team and colleagues developed a high‐content imaging platform to study single cell anticancer drug action in primary patient samples.^10^ Specifically, bone marrow or peripheral blood mononuclear cells (BMMCs or PBMCs) were incubated in non‐adherent monolayers in the presence of small molecule drugs. Putative target cells—primarily neoplastic cells—were identified in high content images based on cell‐surface staining with diagnostic markers. Measuring the drug‐induced on‐target cytotoxicity of cancer cells versus the off‐target cytotoxicity of healthy cells in the same image provided information on a molecule's differential cytotoxicity (Figure S1). This functional analysis of ex vivo drug selectivity has been successfully used to prioritize effective treatments for individual patients in the prospective interventional EXALT‐1 trial.^10^, ^11^

Due to the logistic challenges of fresh sample collection for biomedical research, biobanks of viably frozen material are ever more important to enable timely access to primary tissues for investigators. With this, however, basic questions on sample preparation and storage as well as sample origin have not been adequately addressed. To answer such fundamental questions, we spearheaded a focused program in acute myeloid leukaemia (AML) addressing two aims: (i) Does viable cryopreservation change the cohort responses of target cancer cell cytotoxicity induced by small molecule drugs or have major effects on gene expression? (ii) Does the cohort chemosensitivity change depending on whether material is obtained from the peripheral blood or bone marrow?

To pursue these issues, bone marrow as well as peripheral blood was collected during routine diagnostic procedures from 15 AML patients (Table S1). One half of each of the samples was subjected to functional drug chemosensitivity testing and RNA collection immediately (named “fresh cohort”), the other half was viably biobanked at −196°C based on an established protocol (named “frozen cohort”) (Data S1).^12^, ^13^ A total of 139 small molecule drugs in triplicate at two concentrations with at least 36 DMSO vehicle control wells per plate were analysed for differences in ex vivo chemosensitivity of CD34^+^ cells using PBMCs and BMMCs from fresh and frozen cohorts, respectively. The small molecule library contained FDA approved and investigational drugs and tool compounds against a wide range of targets. Fluorescent drugs were used as quality control metric for the image analysis processing (Table S2).^5^, ^10^ Further, we tested whether cryopreservation had an effect on the transcriptional profiles of CD34^+^ cells in the bone marrow cohort. Comparable cell populations were observed in the cryopreserved cohort upon thawing as compared to the fresh indicating no major background cell loss (data not shown).

The aforementioned high‐content microscopy platform was deployed to quantify on‐target drug action in high content images of BMMCs and PBMCs treated with different small molecule drugs (Data S1). Cancer cell quantification from image analysis correlates strongly with that obtained by flow cytometry (Figure S1) yet offers higher throughput and an approximate 10‐fold reduced cell usage as compared to automated flow cytometry. A further advantage is the ability to leverage robust laboratory automation to measure drug effects of all lineages of cells simultaneously, independent of a cell being adherent or non‐adherent. Ex vivo drug effects for each cohort were quantified by fitting all viable CD34^+^ cell numbers (Figure 1A,C) or cancer cell fractions (Figure 1B,D) after 24 h incubation with each drug at two concentrations as the dependent variable to a generalized linear mixed model using treatment conditions—drug and concentration—as independent predictors. This was done independently for PBMCs versus BMMCs responses (Figure 1A,B) and fresh versus biobanked sample responses (Figure 1C,D). Cohort on‐target differential drug responses were quantified as the coefficient of the respective treatment condition factor and compared to each other.

**FIGURE 1:**
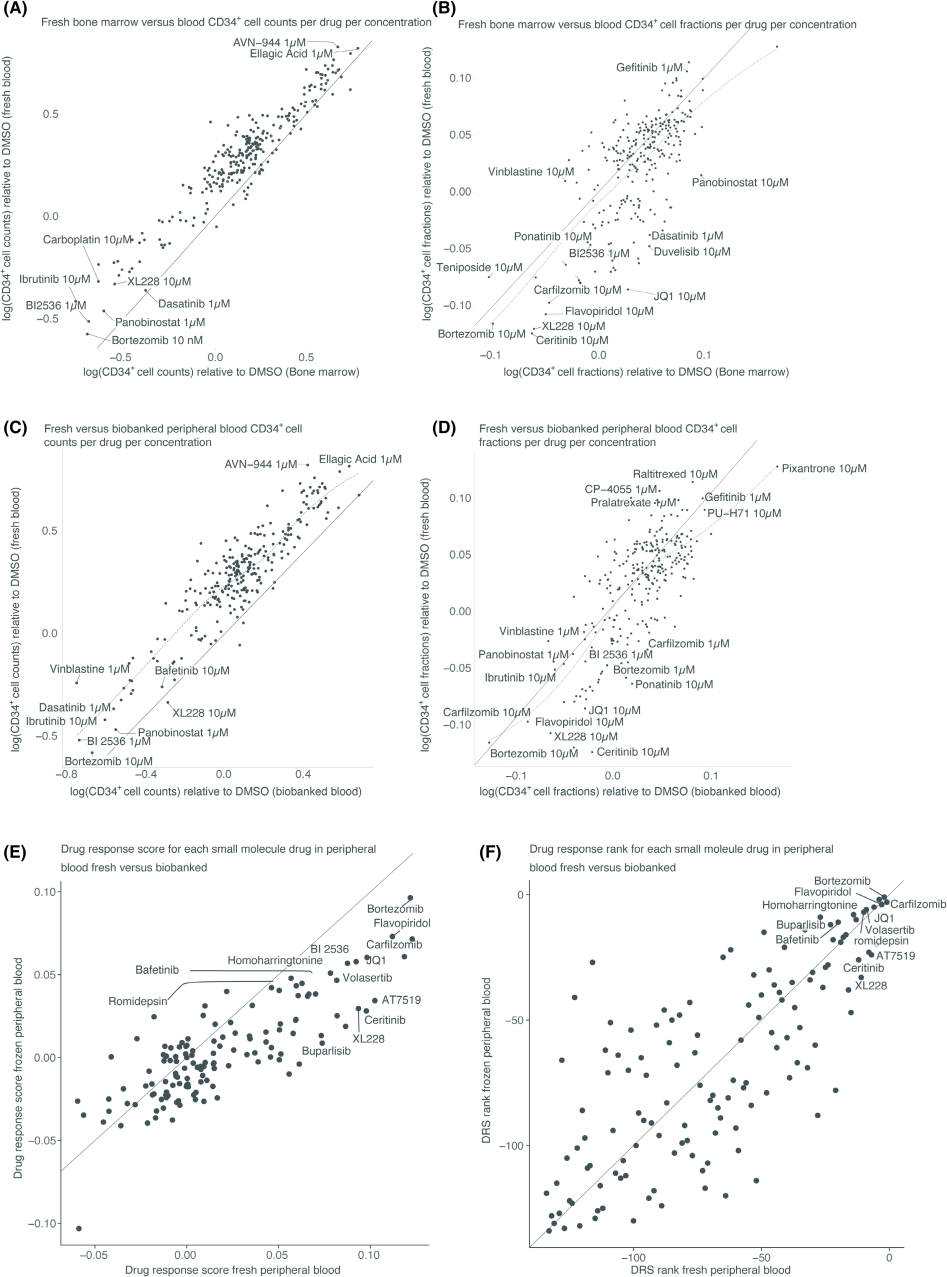
Ex vivo drug responses in fresh versus biobanked AML samples are similar. Coefficients of generalized linear mixed models of (A and C) cancer cell counts or cancer cell fractions (B and D) as a function of drug treatment for fresh bone marrow versus blood (A and B) or biobanked versus fresh material (C and D). Drug response score (E) or ranking (F) of each drug effect on the target population between fresh versus biobanking. No sample data was excluded, all samples for which data were collected are shown, solid line represents x = y, dashed line represents spline fitted trend. Correlation coefficients: (A) *r* = 0.95, (B) *r* = 0.70, (C) *r* = 0.90, (D) *r* = 0.74, (E) *r* = 0.82 (all Pearson) and (D) *r* = 0.77 (Spearman).

The comparison of on target ex vivo drug sensitivity to the 139 small molecules of the CD34^+^ cell fraction revealed a strong correlation between the freshly collected blood and bone marrow sample cohorts for the reduction of cell number and cell fraction (Figures 1A–D). The biobanked cells generally responded slightly stronger to drugs, as did cells derived from the bone marrow indicating that they may be more sensitive. The EXALT‐1 trial ranked drugs by their “drug response score (DRS)” or their ability to selectively target cancer cells over two concentrations.^10^ The higher the DRS ranking, the more effective and targeted the therapy. When using the same analytic method here, the response score and the rank for each drug are similar for the fresh versus biobanked cohort (Figure 1E,F) indicating that biobanked samples could be used for such precision medicine studies, at least in AML, if a delay between sampling and screening is unavoidable.

To determine if any particular pathways were strongly affected by the biobanking process, transcriptomics of enriched CD34^+^ cell populations from both cohorts were compared (Figure 2A,B). Differential expression analysis between the cohorts revealed only a small number of downregulated genes after freezing including genes involved in cell proliferation as well as inflammation, such as FOS and Jun, but none directly relating to major targets of the drug library.

**FIGURE 2:**
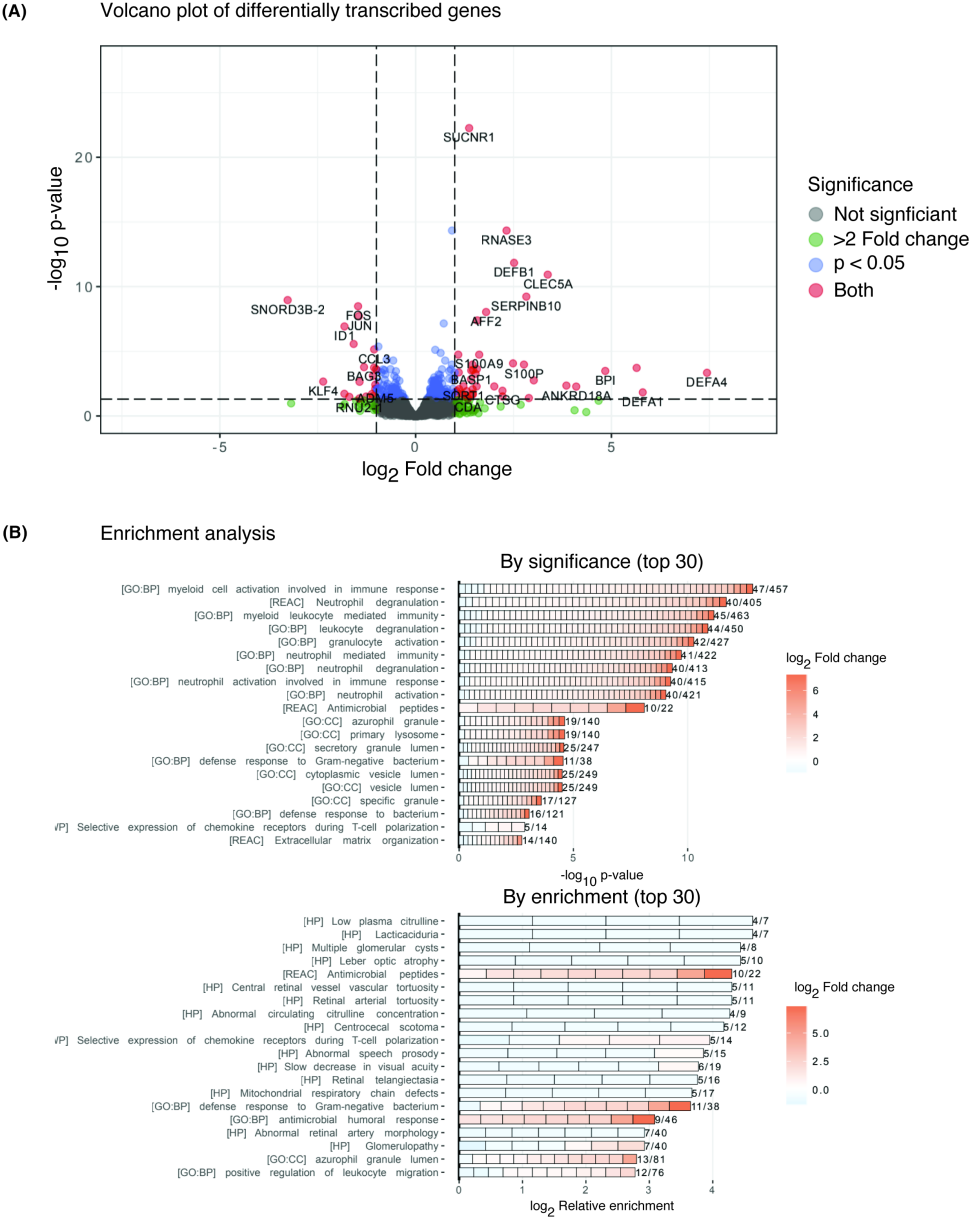
Biobanking does not have profound effects on the transcript of AML cells. Volcano plot of differentially expressed genes in CD34^+^ cells between fresh and biobanked AML samples (A) and differentially expressed pathways based on GO annotations (B).

Together, the results from this pilot study on cohort sample handling for functional testing in AML indicate that logistical challenges with regard to fresh sample access may prospectively be mitigated through the use of professionally maintained biobanks, at least for investigations focused on small molecules and directly cell killing drugs. Some unknowns, however, warrant further investigation: on the one hand, the interplay of cancer and immune cells with immuno‐oncology drugs in the context of reduced inflammation or reduced effector cell activity—on the other, the response of different target cell populations as observed in myelomonocytic or erythroleukaemic subtypes of AML.

## AUTHOR CONTRIBUTIONS

HS recruited patients, collected samples, and ran and oversaw the clinical study. HS, NK drafted the clinical study and NK sponsored the clinical study. NM, KL performed experiments and collected data, NM, NK analysed the single cell response data, RS analysed the RNAseq data, NM, NK, RS, HS, GIV interpreted the data. NK, HS, GIV wrote the manuscript and oversaw the work. All authors approved the manuscript.

## CONFLICT OF INTEREST

NM, BV, RS, NK, GIV, NK and GIV are employees and shareholders of Exscientia. NK and GIV are inventors on patent pending WO2016046346A1 on single cell microscopy and functional drug screening licensed to Exscientia GmbH. All other authors declare no competing interests.

## LICENCE/OTHER

Image analysis software licence and/or data from the image analysis software can be made available by contacting research@exscientia.ai with reasonable requests and collaboration ideas, collaboration is subject to approval.

## Supporting information


Data S1

Table S1

## Data Availability

Level 3 data for this study can be found in the data repository on GitHub under: https://github.com/AC_public/meszaros .

## References

[1] Dietrich S , Oles M , Lu J , Sellner L , Anders S , Velten B , et al. Drug‐perturbation‐based stratification of blood cancer. J Clin Invest. 2018;128(1):427–45. 29227286 10.1172/JCI93801 PMC5749541

[2] van der Velden DL , Hoes LR , van der Wijngaart H , van Berge Henegouwen JM , van Werkhoven E , Roepman P , et al. The drug rediscovery protocol facilitates the expanded use of existing anticancer drugs. Nature. 2019;574(7776):127–31. 31570881 10.1038/s41586-019-1600-x

[3] Montero J , Sarosiek KA , DeAngelo JD , Maertens O , Ryan J , Ercan D , et al. Drug‐induced death signaling strategy rapidly predicts cancer response to chemotherapy. Cell. 2015;160(5):977–89. 25723171 10.1016/j.cell.2015.01.042 PMC4391197

[4] Bhola PD , Ahmed E , Guerriero JL , Sicinska E , Su E , Lavrova E , et al. High‐throughput dynamic BH3 profiling may quickly and accurately predict effective therapies in solid tumors. Sci Signal. 2020;13:eaay1451. 10.1126/scisignal.aay1451 32546544 PMC8023011

[5] Schmidl C , Vladimer GI , Rendeiro AF , Schnabl S , Krausgruber T , Taubert C , et al. Combined chemosensitivity and chromatin profiling prioritizes drug combinations in CLL. Nat Chem Biol. 2019;15(3):232–40. 30692684 10.1038/s41589-018-0205-2 PMC6746620

[6] Pemovska T , Johnson E , Kontro M , Repasky GA , Chen J , Wells P , et al. Axitinib effectively inhibits BCR‐ABL1(T315I) with a distinct binding conformation. Nature. 2015;519(7541):102–5. 25686603 10.1038/nature14119

[7] Bhatt S , Pioso MS , Olesinski EA , Yilma B , Ryan JA , Mashaka T , et al. Reduced mitochondrial apoptotic priming drives resistance to BH3 mimetics in acute myeloid leukemia. Cancer Cell. 2020;38(6):872–890.e6. 33217342 10.1016/j.ccell.2020.10.010 PMC7988687

[8] Haibe‐Kains B , El‐Hachem N , Birkbak NJ , Jin AC , Beck AH , Aerts HJ , et al. Inconsistency in large pharmacogenomic studies. Nature. 2013;504(7480):389–93. 24284626 10.1038/nature12831 PMC4237165

[9] Krall N , Superti‐Furga G , Vladimer GI . Patient‐derived model systems and the development of next‐generation anticancer therapeutics. Curr Opin Chem Biol. 2020;01(56):72–8. 10.1016/j.cbpa.2020.01.002 32086157

[10] Snijder B , Vladimer GI , Krall N , Miura K , Schmolke AS , Kornauth C , et al. Image‐based ex‐vivo drug screening for patients with aggressive haematological malignancies: interim results from a single‐arm, open‐label, pilot study. Lancet Haematol. 2017;4(12):e595–606. 29153976 10.1016/S2352-3026(17)30208-9 PMC5719985

[11] Kornauth C , Pemovska T , Vladimer GI , Bayer G , Bergmann M , Eder S , et al. Functional precision medicine provides clinical benefit in advanced aggressive hematologic cancers and identifies exceptional responders. Cancer Discov. 2022;12(2):372–87. 34635570 10.1158/2159-8290.CD-21-0538 PMC9762339

[12] Lal R , Lind K , Heitzer E , Ulz P , Aubell K , Kashofer K , et al. Somatic TP53 mutations characterize preleukemic stem cells in acute myeloid leukemia. Blood. 2017;129(18):2587–91. 28258055 10.1182/blood-2016-11-751008

[13] Caraffini V , Geiger O , Rosenberger A , Hatzl S , Perfler B , Berg JL , et al. Loss of RAF kinase inhibitor protein is involved in myelomonocytic differentiation and aggravates RAS‐driven myeloid leukemogenesis. Haematologica. 2020;105(2):375–86. 31097632 10.3324/haematol.2018.209650 PMC7012480

