# Supplementary material for: Influence of cryopreservation on drug responses and gene expression of AML cells: Implications for the use of biobanked tissues
Source: Br J Haematol. 2022 Nov 10;200(3):e32–6. doi: 10.1111/bjh.18557 (PMC10100012; doi:10.1111/bjh.18557)
Supplement: Supplementary file 1 — Data S1 [file BJH-200-e32-s002.docx]

Supplementary information

**Influence of cryopreservation on drug responses and gene expression of AML cells: implications for the use of biobanked tissues**

Noemi Meszaros ^1*^, Karin Lind ^2^, Robert Sehlke ^1*^, Bojan Vilagos ^1*^, Nikolaus Krall ^1*^, Gregory I. Vladimer ^1*^, and Heinz Sill ^2^

^1^ Exscientia, Campus-Vienna-Biocenter 5, 1030 Vienna, Austria

^2^ Division of Hematology, Medical University of Graz, Auenbruggerplatz 38, 8036 Graz, Austria

**Supplementary methods**

*Patient recruiting, sampling, and cell purification/ cell selection*

The study was approved by the ethics committee of Medical University of Graz, Graz, Austria (MUG) and samples were collected after written informed consent of each patient. Inclusion criteria included: a) patient age of 18 years or older at the time of signing the informed consent form; b) newly diagnosed primary, secondary, or therapy-related AML other than M3; relapsed AML; c) population of >20% leukemic blast cells in the bone marrow; d) stabilizer-free heparin used as anticoagulant; e) patient material frozen at the leukemia biobank of MUG. Exclusion criteria were as follows: a) diagnosis acute promyelocytic leukemia; b) unavailability of fresh and viable material containing AML blast cells; c) patient undergoing radiation treatment for a concomitant disorder within 2 weeks prior to sampling; d) patient already undergoing AML-specific chemotherapy; e) known pregnancy. Clinical data of the patients analyzed in this study are depicted in the Supplementary Table 1.

An outline of the experimental procedure is depicted in the Supplementary Figure 1. Mononuclear cells from bone marrow and/or peripheral blood samples were prepared using a Ficoll density gradient (GE Healthcare, Solingen, Germany) following manufacturer´s directions. The cells were subjected either immediately to high-content image analysis of 139 small molecule drugs (Supplementary Table 2) or RNA extraction for RNA sequencing (RNAseq), or viability cryopreserved in 10% DMSO in fetal bovine serum and stored in liquid nitrogen for 1-3 months until use.

*Flow cytometry*

Following mononuclear cell isolation, cells were resuspended in flow cytometry buffer (DPBS + 5% FBS) and incubated with CD34, CD117, and CD33 (all BD Biosciences, Heidelberg, Germany) fluorescent antibodies. During the entire procedure the cells were kept on ice. Samples were analysed using the FACSVia flow cytometry system (BD Biosciences) and the BD FACSVia Research Software.

*Imaging and analysis*

Imaging of the primary cell monolayer was performed using PerkinElmer CLS spinning disk automated confocal microscopes (PerkinElmer, Rodgau, Germany), with non-overlapping, sequential, fluorescent channel imaging. All images were taken with a 10x objective. Four fields were imaged, representing at least 50% of the well bottom, for each well. For analysis, images are first subjected to illumination correction and proprietary pre-processing. Cell segmentation and classification deployed for this project detects cells by first detecting the nuclei and then distinguishing between viable or non-viable and marker positive or marker negative cells using specialized convolutional neural networks that were trained with manually annotated images from a wide variety of primary human cancer samples. Notably, the standard of care AML drug daunorubicin was excluded from this analysis due to its fluorescent nature, as well as other fluorescent drugs. Drug response was quantified for blood or bone marrow under fresh or biobanked conditions separately by fitting viable CD34+ cell numbers or cell fractions to generalized linear models using treatment conditions as the predictors as outlined in the main body text. A logarithmic link function was used. The coefficients for each drug treatment and concentration were plotted against each other to compare results under different conditions. The Drug Response Score (DRS) was calculated as described previously (*Snijder B et al. Lancet Haematol 2017;4:e595-e606*) and plotted directly for different conditions. Pearson and Spearman correlation coefficients were calculated.


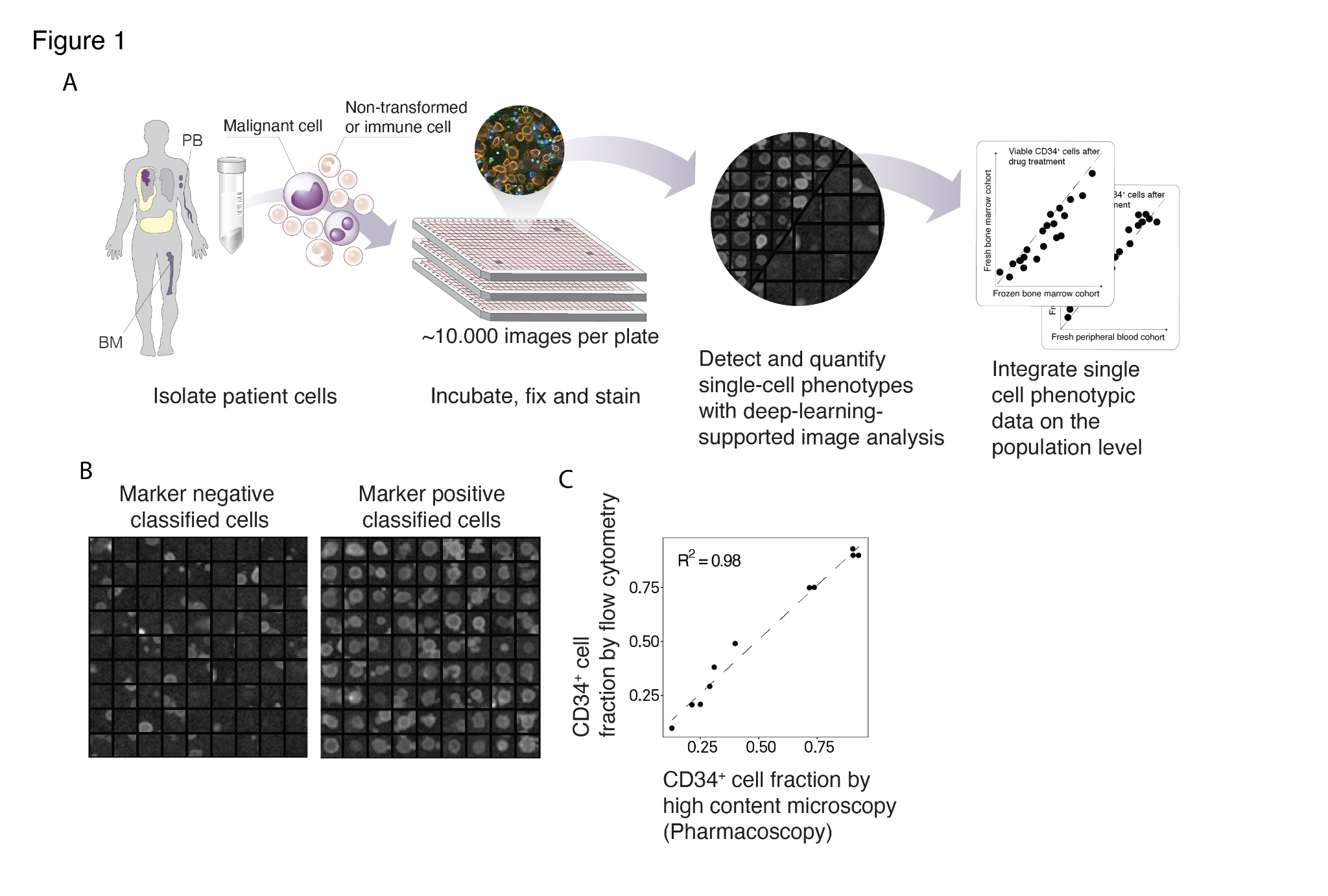


**Supplementary Figure 1. Functional drug screening by high-content microscopy.** (A) An overview of the employed functional drug screening pipeline; (B) example cell classification of 100 CD34^+^ cells into positive and negative groups through machine learning; (C) flow cytometry analysis compared to high-content detection, representing all samples analyzed.

**Supplematary Table 2. Small molecule drugs used in the study**

**----------------------------------------------------------------------------------------------------**

Azacitidine

Pemetrexed

Ellagic Acid

Celecoxib

Erlotinib

Hydroxyurea

Mitomycin C

Methotrexate

Temsirolimus

Tacedinaline

Belinostat

Bafetinib

Cladribine

Pentostatin

Gemcitabine

Docetaxel

AVN-944

Doxorubicin

Midostaurin

Lomustine

ABT-263 = Navitoclax

Lestaurtinib

Decitabine

Everolimus

Sorafenib

Carfilzomib

Etoposide

Sunitinib

Ibrutinib

Imatinib

Valrubicin

Camptothecin

Tamibarotene

Topotecan

ATRA

Clofarabine

Nilotinib

Thiotepa

AT9283

Fluorouracil

Pomalidomide

Vorinostat

Pazopanib

Carboplatin

Pivaloyloxymethyl butyrate

Tacrolimus

Dabrafenib

dBET6

Rapamycin

Methylprednisolone

Duvelisib

Vinblastine

Cytarabine

Romidepsin

Ifosfamide

Pralatrexate

BRD4-MTX_1

Thioguanine

Bortezomib

Prednisolone

Selinexor (KPT-330)

Mercaptopurine

Dexamethasone

MGCD-0103

Melphalan

Troxacitabine

Palbociclib

Paclitaxel

Ixabepilone

Thalidomide

Crizotinib

Bendamustin

Carmustine

Flutamide-phenprocoumon

Homoharringtonine

XL228

Pixantrone

EGCG = Epigallocatechin gallate

CHR-2797

Temozolomide

Rebastinib

Vindesine

Lenalidomide

AC220 = Quizartinib

dCDK6

BEZ235

Daunorubicin

Capecitabine

Buparlisib (BKM120)

Bexarotene

Cyclophosphamide

CP-4055

Atorvastatin

TAF1_1

Teniposide

AT7519

Zoledronate

Busulfan

Ruxolotinib

Prednisone

MI-3-89

Mitoxantrone

Idarubicin

Crenolanib

Chlorambucil

ABT-199 = Venetoclax

Ceplene

Vandetanib

Cyclosporine

Gefitinib

BI 2536

TAF1_2

Leflunomide

Volasertib

Dasatinib

Idelalisib

Vincristine

MLN-518 = Tandutinib

PU-H71

Cisplatin

Raltitrexed

Obatoclax

Ceritinib

ABT-869 = Linifanib

dBRD9

JQ1

Panobinostat

Masitinib

Ponatinib

Nelarabine

Cabozantinib

Amsacrine

Bosutinib

CHIR-258

Flavopiridol

R115777

BRD4-MTX_2

ABT-737

Fludarabine

*RNA extraction and sequencing analysis*

CD34^+^ cells were extracted using EasySep™ Human CD34 positive selection, (Stemcell Technologies, Vancouver, Canada) following manufacturer´s directions and purification confirmed by flow cytometry. RNA extraction was performed using a Qiagen RNeasy mini kit (Qiagen, Hilden, Germany) following manufacturer´s directions. WuxiNextCode performed the sequencing and standard analysis using the following metrics: RNA concentration was measured by NanoDrop, and RNA quality is evaluated by Agilent 2100 Bioanalyzer (Agilent Technologies, Santa Clara, CA). Library preparation was done with a TruSeq Stranded Total RNA Gold Library Prep Kit with RiboZero treatment, and paired end 150bp sequencing was performed on a HiSeq X platform (Illumina, San Diego, CA). The pre-processing pipeline included adaptor trimming: Skewer v0.2.1 (FFPE only), alignment: STAR v2.5.3a, SAMtools v0.1.19 (sorting/indexing), transcript quantification: RSEM v1.3.0, and QC Metrics: FASTQC v0.11.3. A reference genome of hg19/hg38 was used.
